# First branchial cleft cysts in a UK paediatric tertiary centre: A 10-year single-centre case series

**DOI:** 10.1007/s00383-026-06298-6

**Published:** 2026-02-09

**Authors:** Sofia Anastasiadou, Paris Bruno, Oliver Dale, Julian Gaskin

**Affiliations:** Aristotle University of Thessaloniki, Thessaloniki, Greece

**Keywords:** First branchial cleft anomaly, Paediatric, Congenital neck lesions, Branchial cleft sinus, Branchial cleft fistula, Facial nerve, Parotid gland, Magnetic resonance imaging, Surgical excision, Postoperative complications

## Abstract

**Background:**

First branchial cleft anomalies are uncommon paediatric congenital lesions that may present as persistent pre-auricular or post-auricular sinuses and can involve the parotid region and facial nerve. We present a single-centre 10-year experience describing presentation, imaging, management and outcomes.

**Methods:**

Retrospective review of a prospectively collected local dataset of paediatric patients with first branchial arch cleft cysts/tracts who underwent evaluation and surgery at a tertiary paediatric centre. Data elements included age at presentation, presenting symptoms, imaging modality, surgical treatment, postoperative complications, facial nerve dysfunction, fistula course, recurrence and follow-up.

**Results:**

Eleven patients were included (mean age 5.55 years, median 4 years). The commonest presentation was a persistent sinus with purulent discharge (9/11). Laterality was predominantly left-sided (8/11). MRI was the most used imaging modality (8/11). Postoperative wound infection occurred in 3/11 patients (27%); marginal mandibular branch weakness was recorded in 2/11 (both documented as transient/resolved). One patient had documented recurrence. The fistula/tract most commonly tracked from level II to the ear canal (9/11).

**Conclusion:**

In this paediatric series, first branchial arch cleft anomalies most commonly presented as persistent left-sided sinuses with purulent discharge. MRI was commonly used for preoperative assessment. Surgical excision was associated with wound infection in a minority and transient marginal mandibular weakness in several cases. Larger multi-centre series with systematic prospective follow-up are needed.

## Introduction

First branchial cleft anomalies represent an uncommon group of congenital malformations arising from incomplete obliteration of the first branchial cleft during embryogenesis. Although anomalies of the second branchial cleft are more frequently encountered in clinical practice, first branchial cleft lesions are rare and often misdiagnosed [1]. They may present as cysts, sinuses, or fistulae, and are most frequently located in the periauricular or parotid regions, extending towards the external auditory canal, the pre-auricular area, or the angle of the mandible. The close anatomical relationship between these anomalies, the parotid gland, and the branches of the facial nerve makes their recognition and management clinically important and technically challenging [2].

The present study describes the 10-year experience of a single UK tertiary paediatric otolaryngology centre in the diagnosis and management of first branchial cleft anomalies. The study aims to characterise presentation patterns, imaging strategies, operative findings, complications, and outcomes in this patient group. By presenting detailed observational data, we seek to highlight the practical challenges of diagnosis and management and to situate our findings within the context of the wider literature.

## Methods

This study is a retrospective case series based on a prospectively maintained local dataset. The cohort comprised all paediatric patients who underwent evaluation and surgery for first branchial cleft anomalies at a single UK tertiary centre over a ten-year period. Inclusion criteria were the presence of a first branchial arch cyst, sinus, or fistula confirmed clinically and intraoperatively. All patients were younger than 18 years old, otherwise fit and well with no other craniofacial syndromes, operation or anomalies. Patients with incomplete data due to missing records were excluded.

Patient data were extracted from clinical records, operative notes, and imaging reports. Variables included age at presentation, clinical presentation, laterality, imaging modality, fistula course, surgical details, postoperative complications, facial nerve function, recurrence, and duration of follow-up.

Age at presentation was recorded in years. Presenting symptoms were captured verbatim from records and grouped into descriptive categories which are purulent sinus discharge or swelling. Laterality was not consistently documented as a discrete field and was therefore inferred from descriptive free text or retrieved from the operation note. Imaging data were extracted according to the modality used, including ultrasound and magnetic resonance imaging (MRI). Operative findings included the anatomical course of the fistula or sinus tract, described relative to neck levels and the external auditory canal. Work classification was used to characterise the course further.

Postoperative complications were identified from operative and clinic documentation, including wound infection, marginal mandibular branch weakness, or other sensory or motor changes. Facial nerve dysfunction was analysed separately where documented, and if resolution was described in follow-up notes, the deficit was categorised as transient. Recurrence was recorded if explicitly stated; where no information was available the field was marked as missing or unknown.

Descriptive statistics were generated, including means, medians, and ranges for continuous data and proportions for categorical data. Inferential statistical testing was not attempted because of the small sample size and incomplete dataset.

Institutional approval was obtained from the hospital’s audit and clinical governance department. Patient-level identifiers were removed prior to analysis.

## Results

**Fig. 1:**
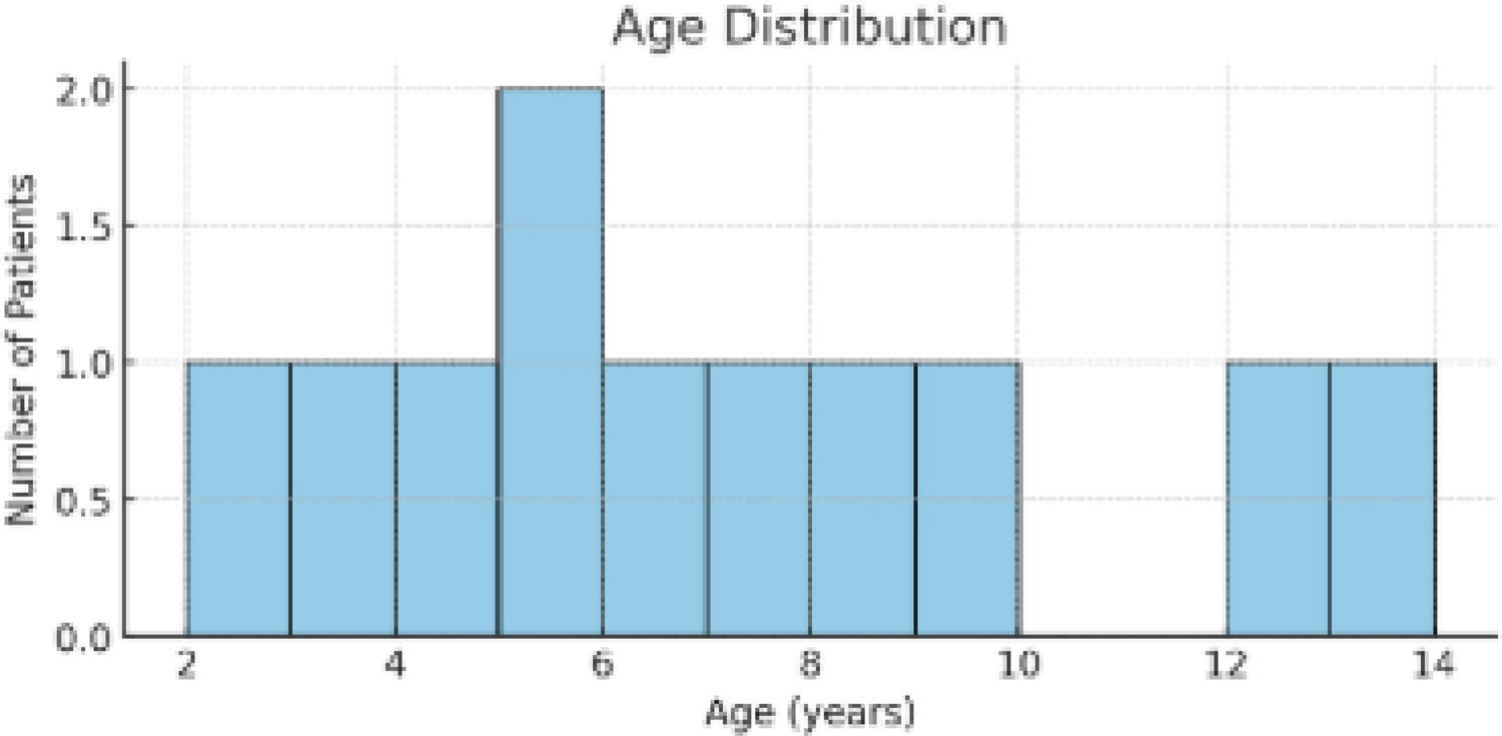
Age distribution

A total of eleven patients met inclusion criteria over the ten-year period. The mean age at presentation was 5.55 years, with a median of four years and a range from two to thirteen years (Fig. 1). All results are summarised in Tables 1 and 2.

**Fig. 2:**
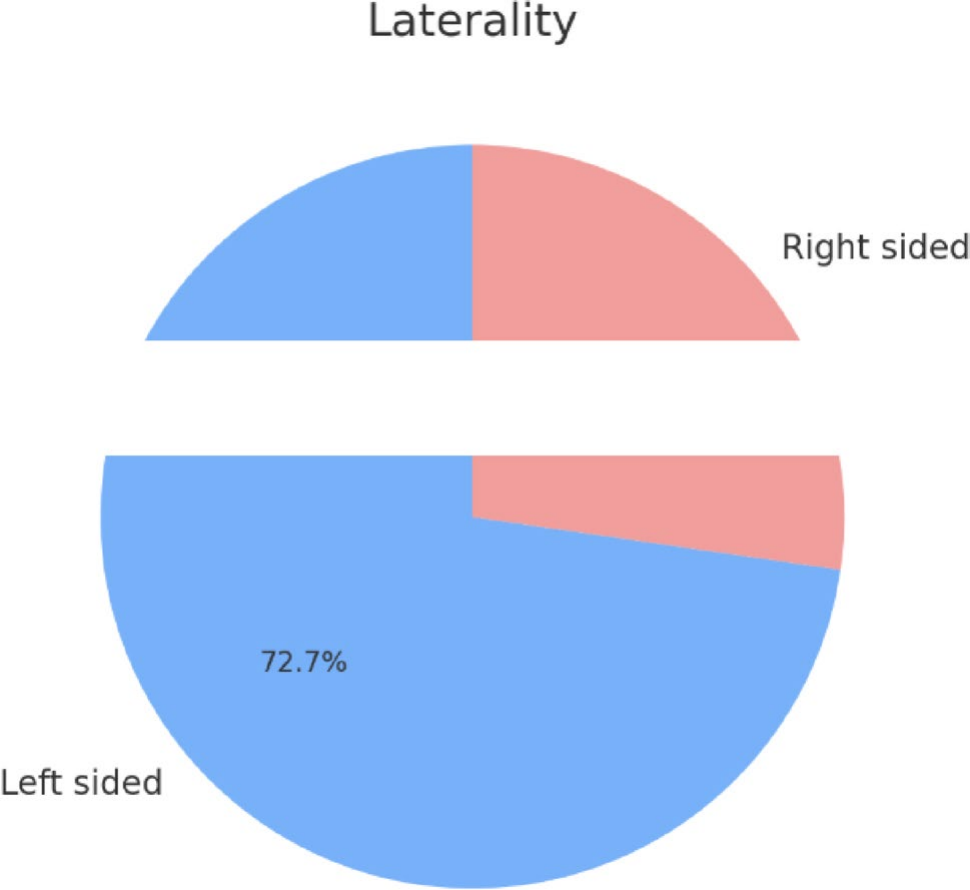
Laterality of lesion

Laterality was predominantly left, with 8/11 to be left-sided and the rest right sided (Fig. 2).

**Fig. 3:**
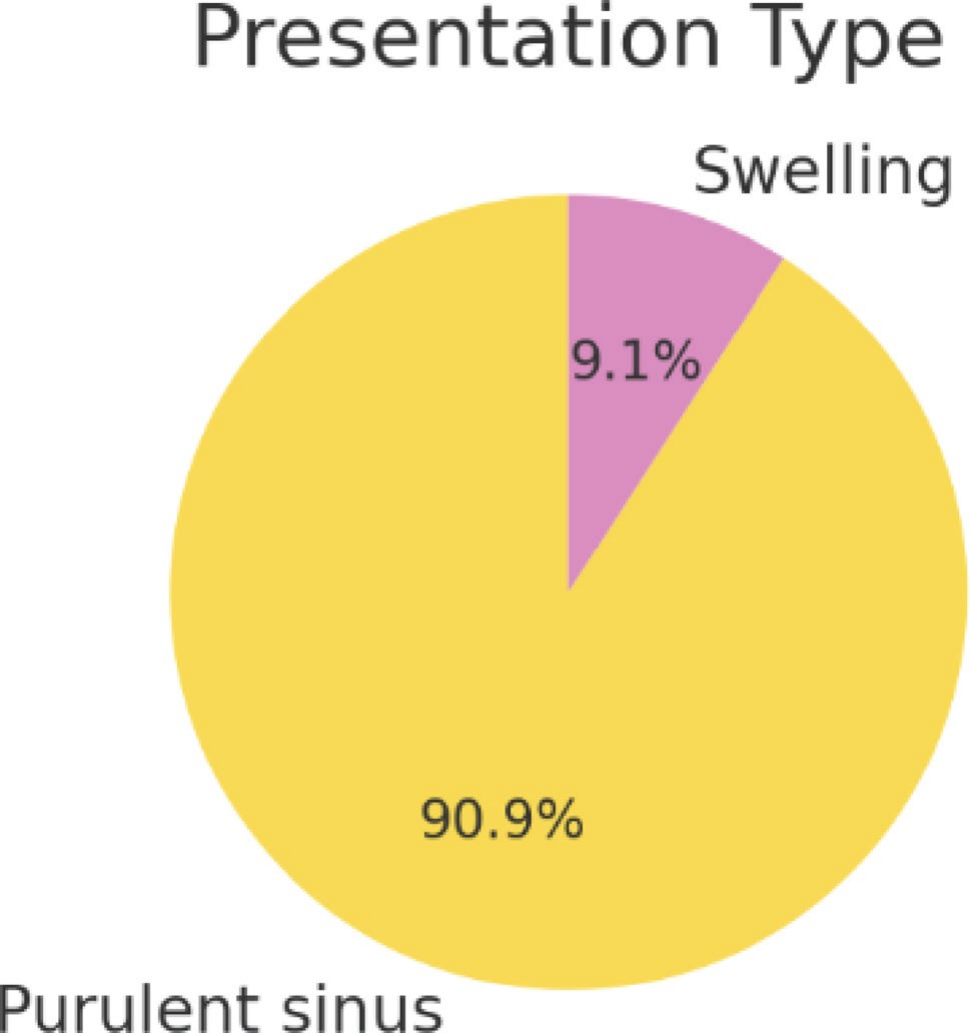
Presentation symptoms

In terms of clinical presentation, persistent sinus was the most common manifestation, documented in ten of eleven patients (91%) (Fig. 3). Nine of these presented with purulent discharge, most commonly arising at the angle of the mandible or in the pre-auricular region. One patient presented with swelling alone without a discharging sinus.

**Fig. 4:**
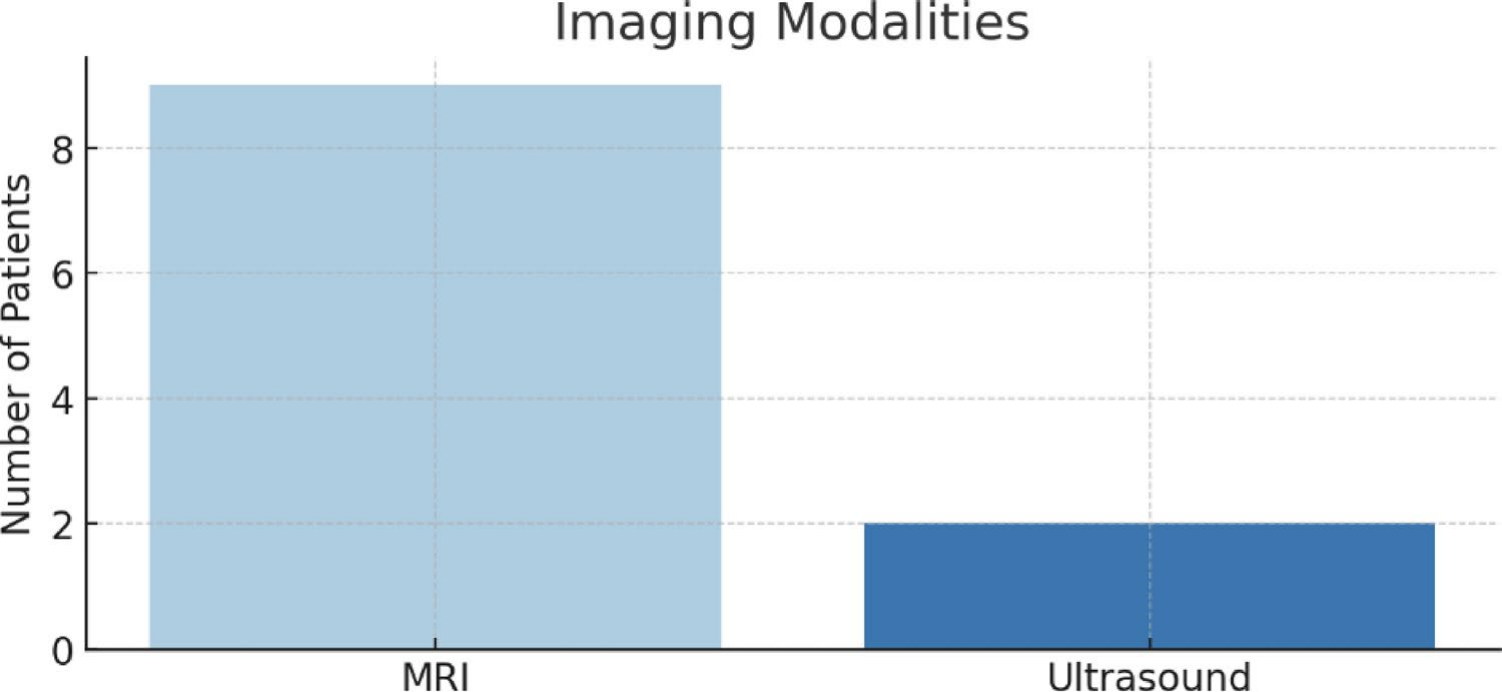
Imaging modalities

MRI was the imaging modality most frequently used, undertaken in eight of eleven patients (73%) (Fig. 4). Two patients underwent ultrasound imaging, and one patient had two separate MRI scans during the diagnostic process. MRI was generally favoured for its ability to delineate tract anatomy and its relationship to the parotid and facial nerve. USS was only used to diagnose the pathology between the years 2015–2017 a fact that possibly reflects shifting to a more detailed imaging modality such as an MRI in the more recent years.

**Fig. 5:**
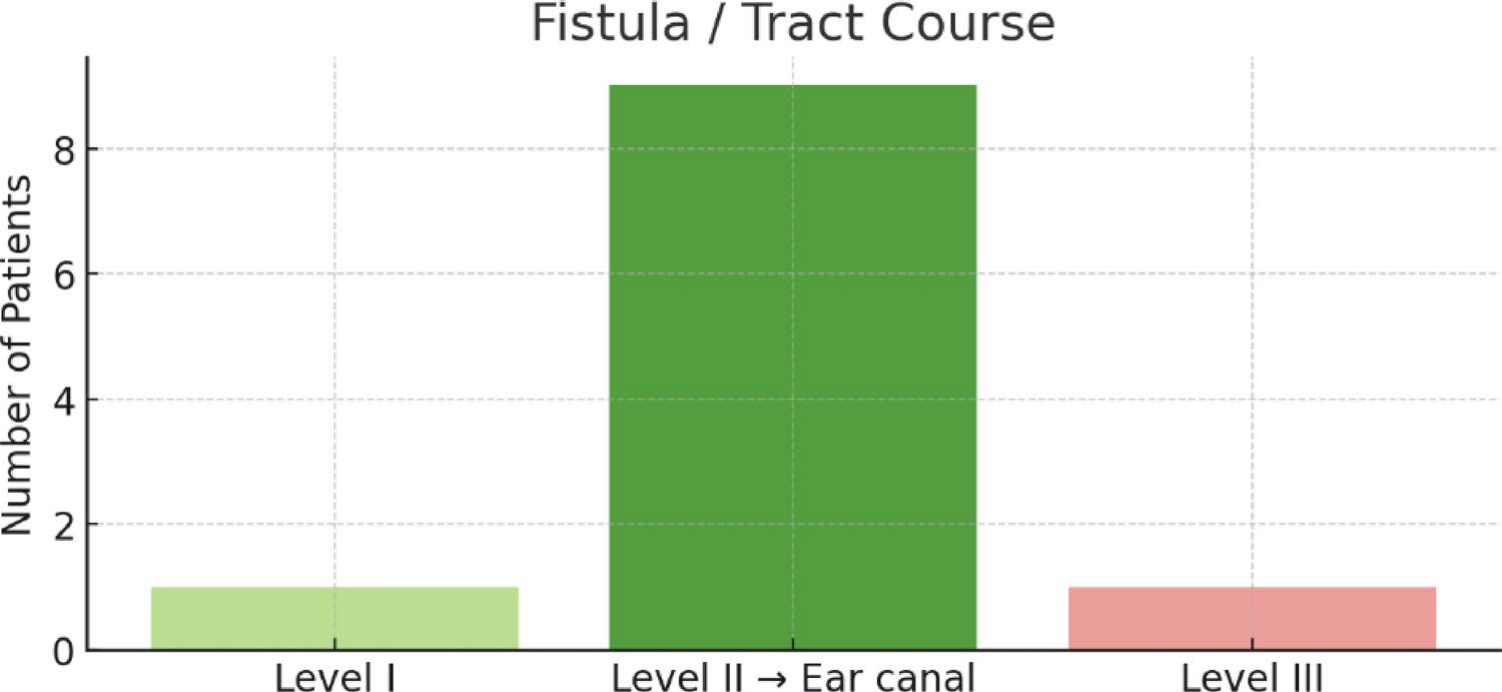
Fistula course

Intraoperative findings revealed that the tract or fistula most commonly extended from level II of the neck to the external auditory canal, observed in nine of eleven cases (82%) (Fig. 5). One tract coursed from level I, and another from level III. These findings underscore the frequent involvement of the parotid region and the potential risk to the facial nerve.

**Fig. 6:**
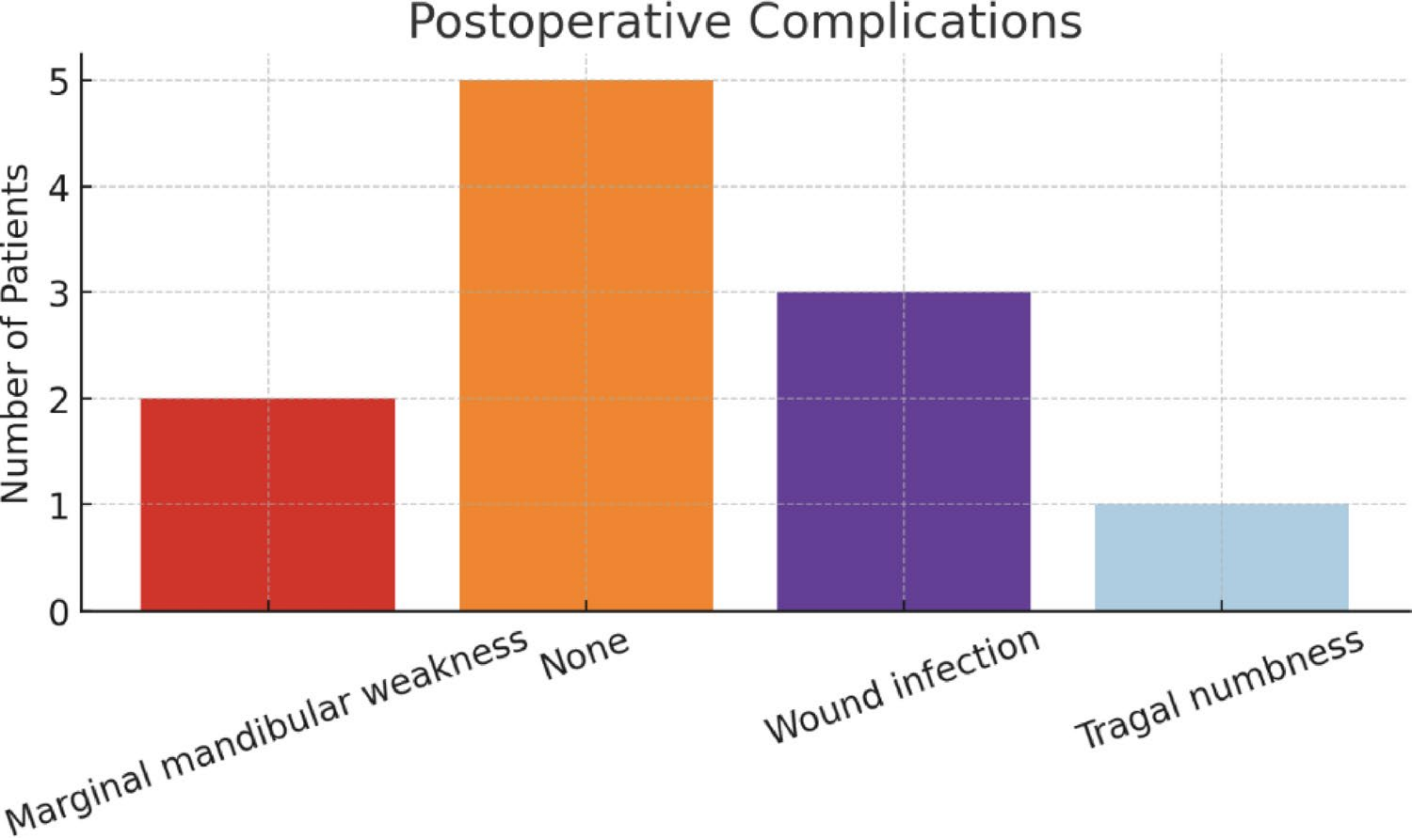
Post-operative complications

Postoperative outcomes varied. Five patients experienced no complications following surgery. Wound infection was documented in three cases (27%) (Fig. 6). These patients were treated with oral antibiotics and were followed up until complete resolution of symptoms. Marginal mandibular branch weakness was observed in two patients (18%), both of whom demonstrated full recovery during follow-up. One patient reported tragal numbness postoperatively.

Pre-operative, intra-operative and post-operative examples of one of our cases are presented in Figs. 7, 8, 9, 10 and 11 and MRI imaging of the same case is presented in Figs. 12, 13, 14, 15 and 16.

**Fig. 7:**
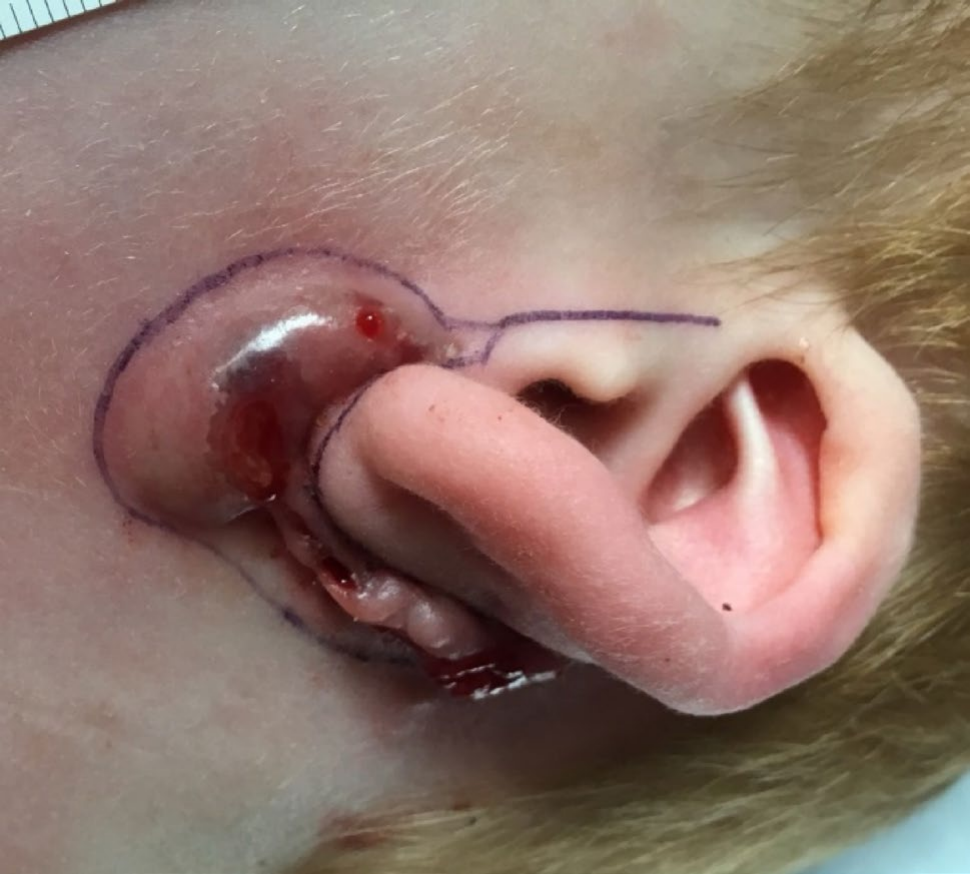
Pre-operative appearances - marking

**Fig. 8:**
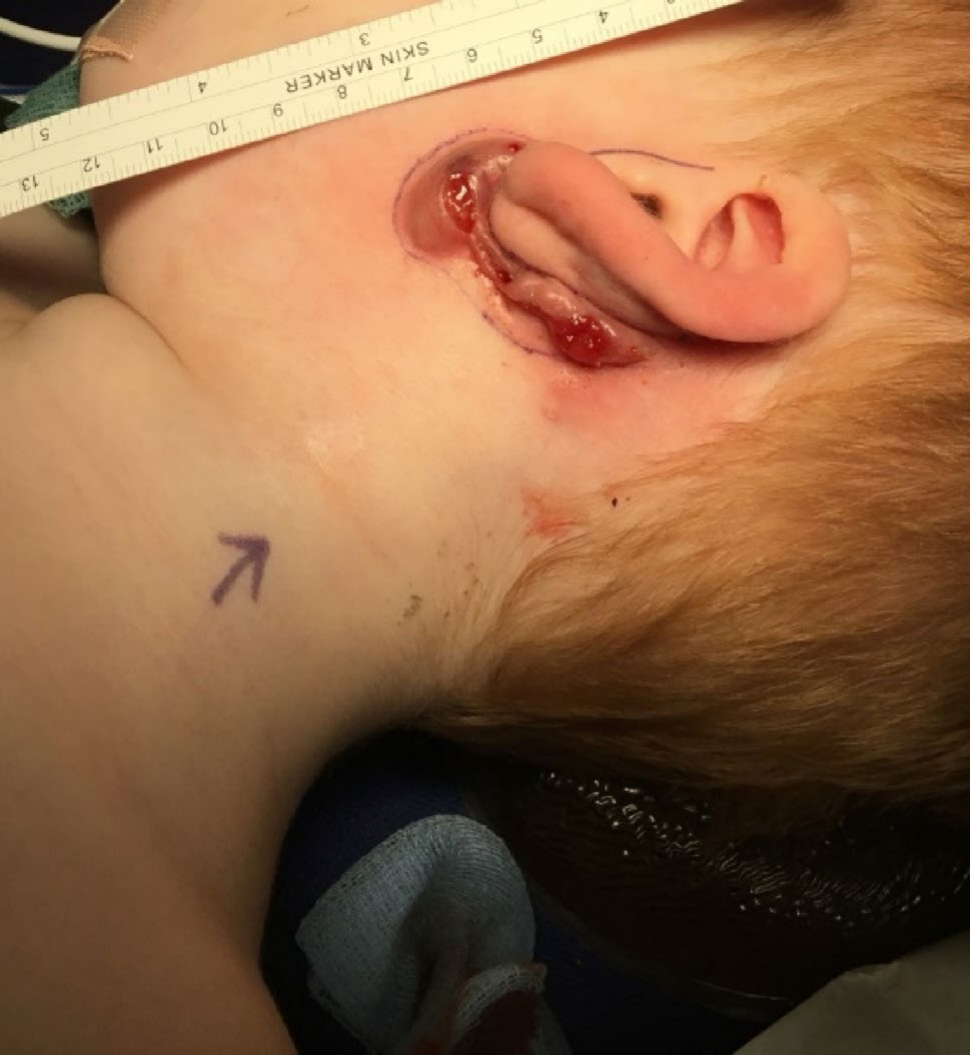
Pre-operative appearances - marking

**Fig. 9:**
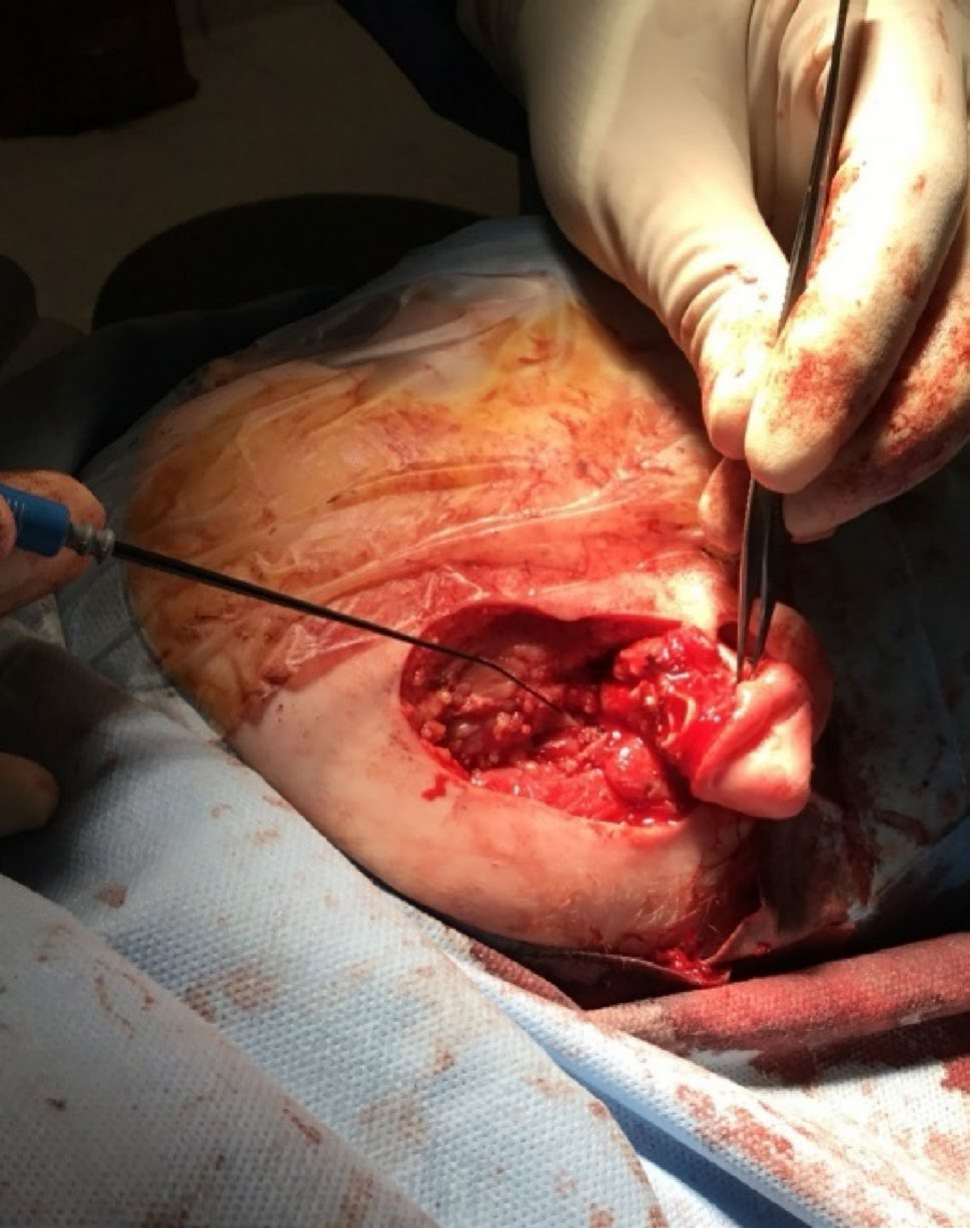
Intraoperative appearances - Facial nerve is indicated

**Fig. 10:**
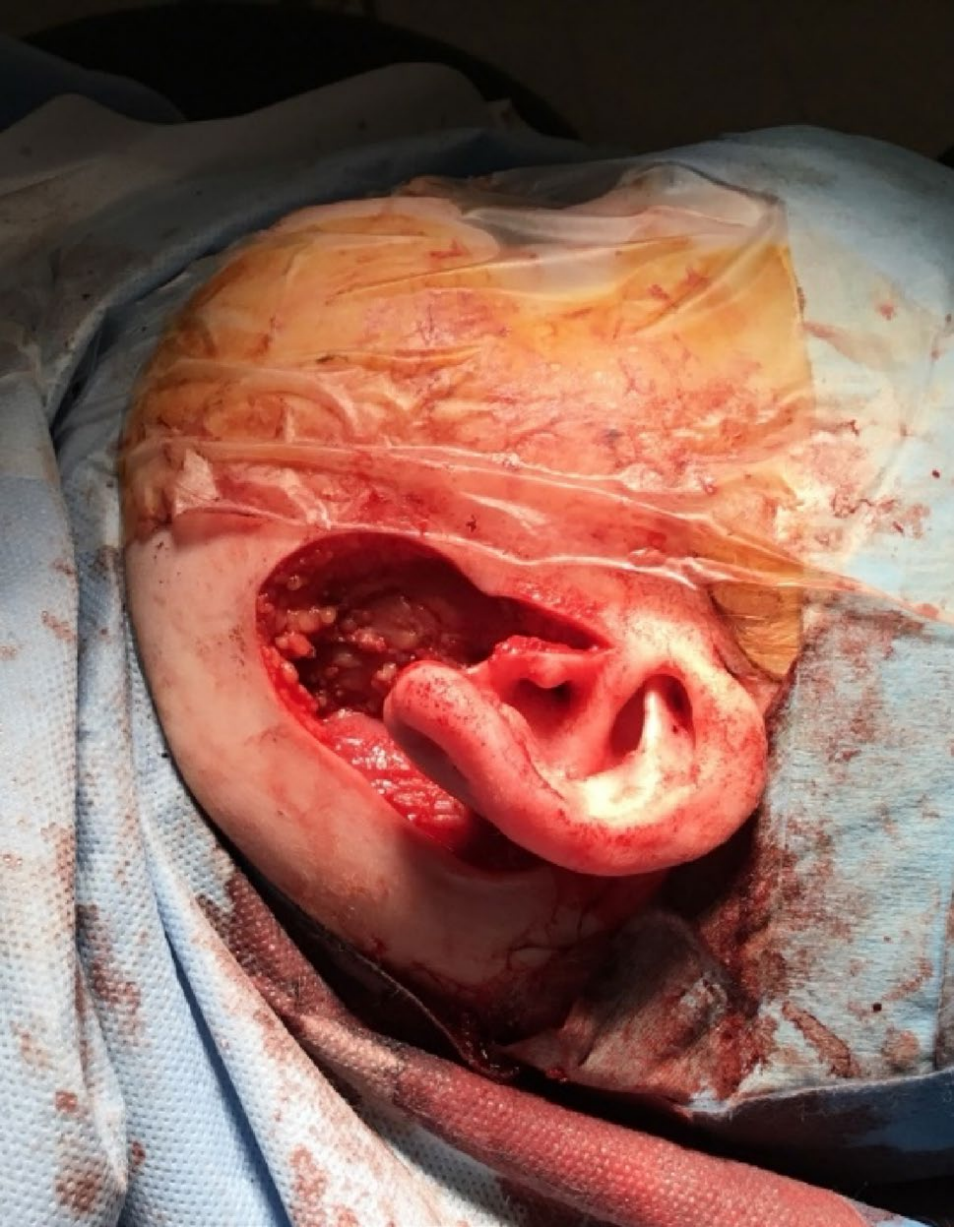
Intra-operative appearences

**Fig. 11:**
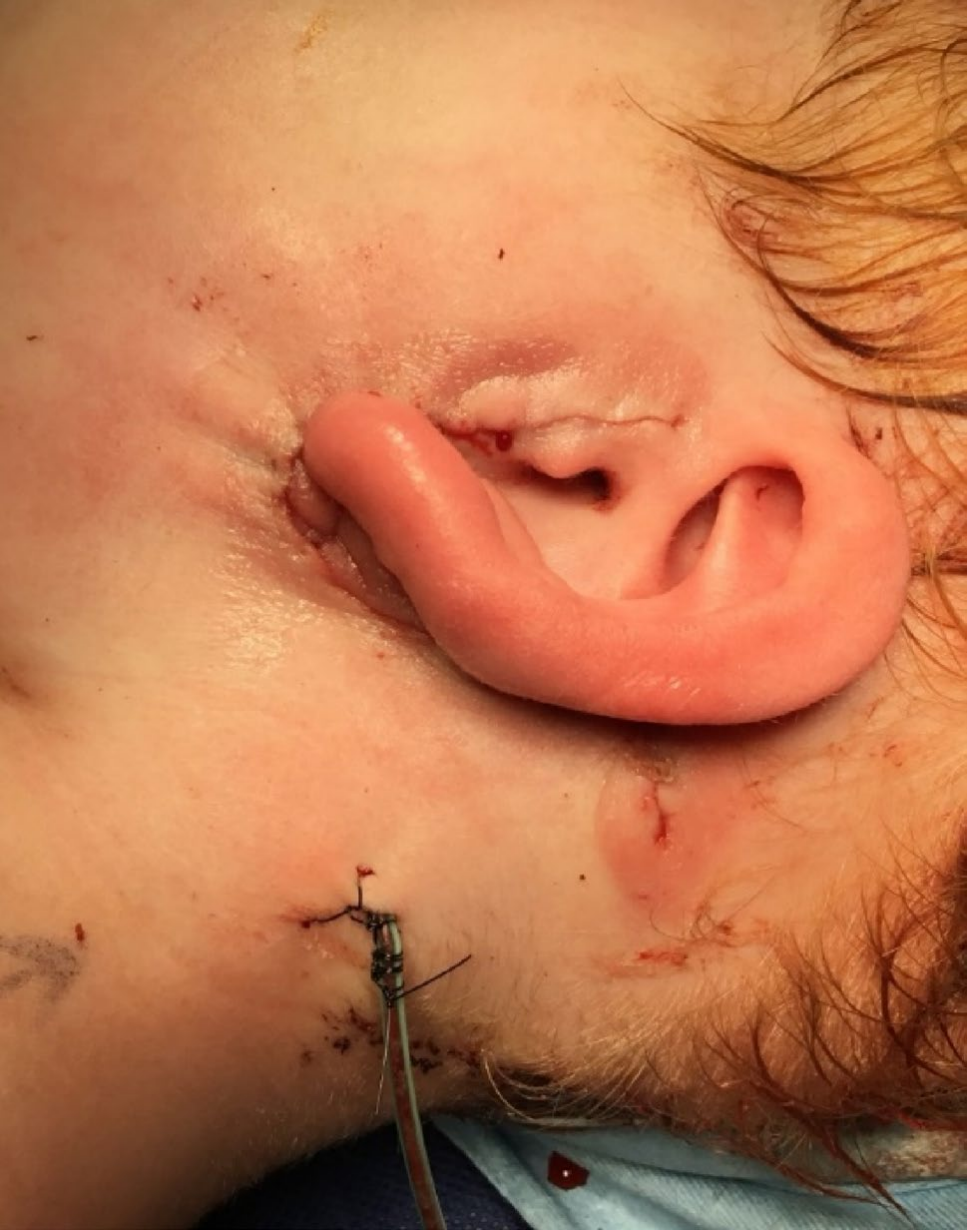
Post-operative appearances - skin closure

**Fig. 12:**
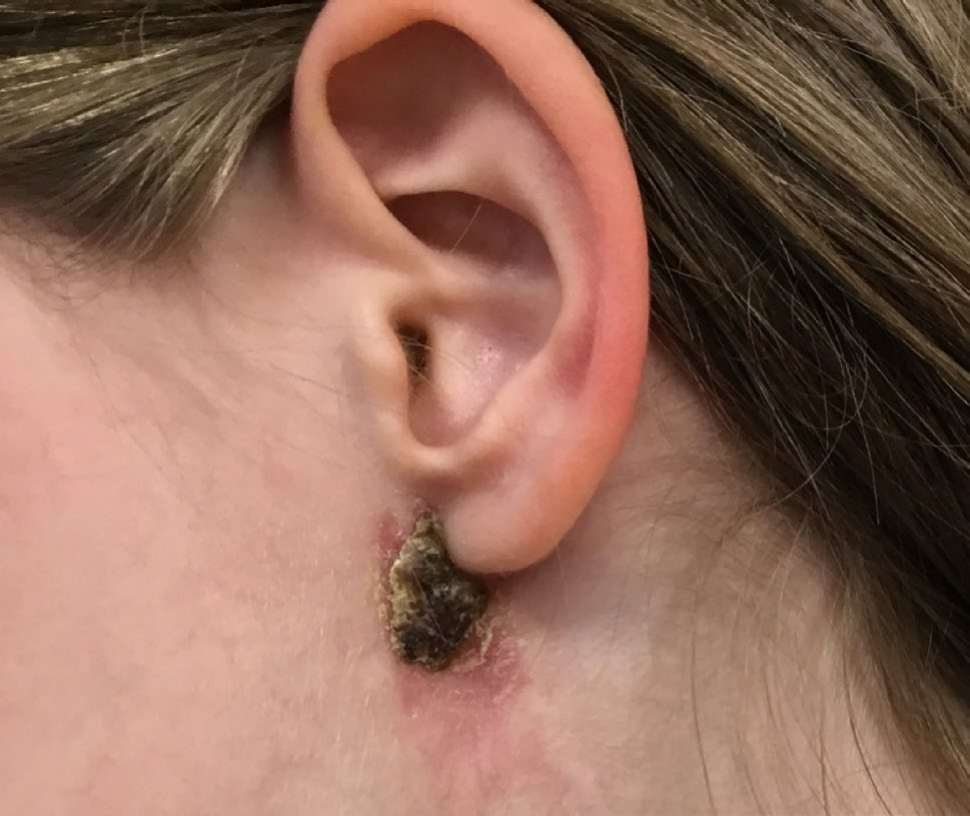
Recurrence presentation

**Fig. 13:**
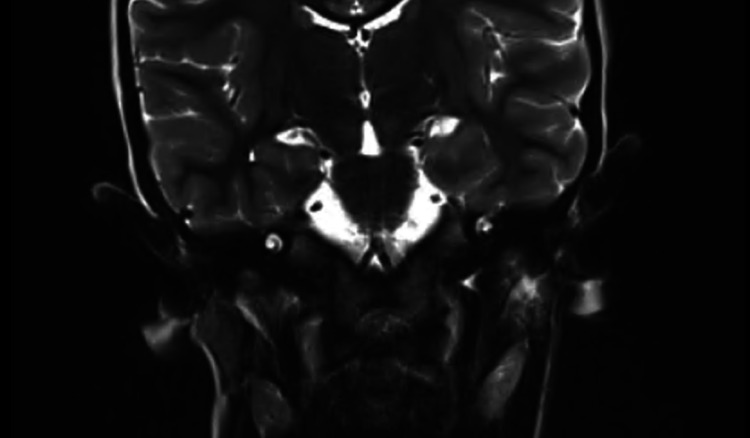
MRI imaging of post-operative recurrence on the left side – coronal view

**Fig. 14:**
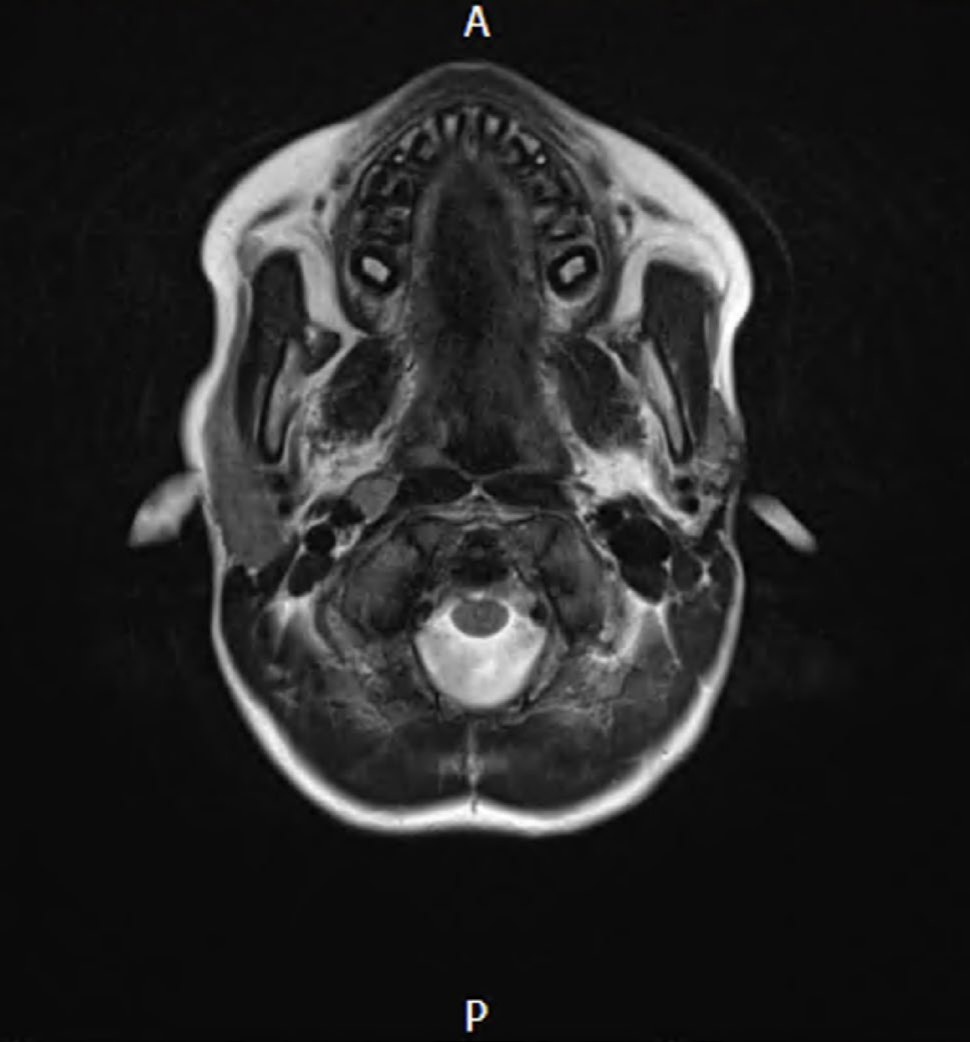
MRI imaging of post-operative recurrence on the leftside – axial view

Recurrence was documented in one patient (Fig. 12). The MRI of the recurrence case is also presented in Figs. 13 and 14. This patient was referred to another tertiary paediatric ENT unit with specialisation in head and neck surgery. The child had a revision excision and was fully treated. They were followed up for 6 months subsequently to ensure complete resolution.

**Fig. 15:**
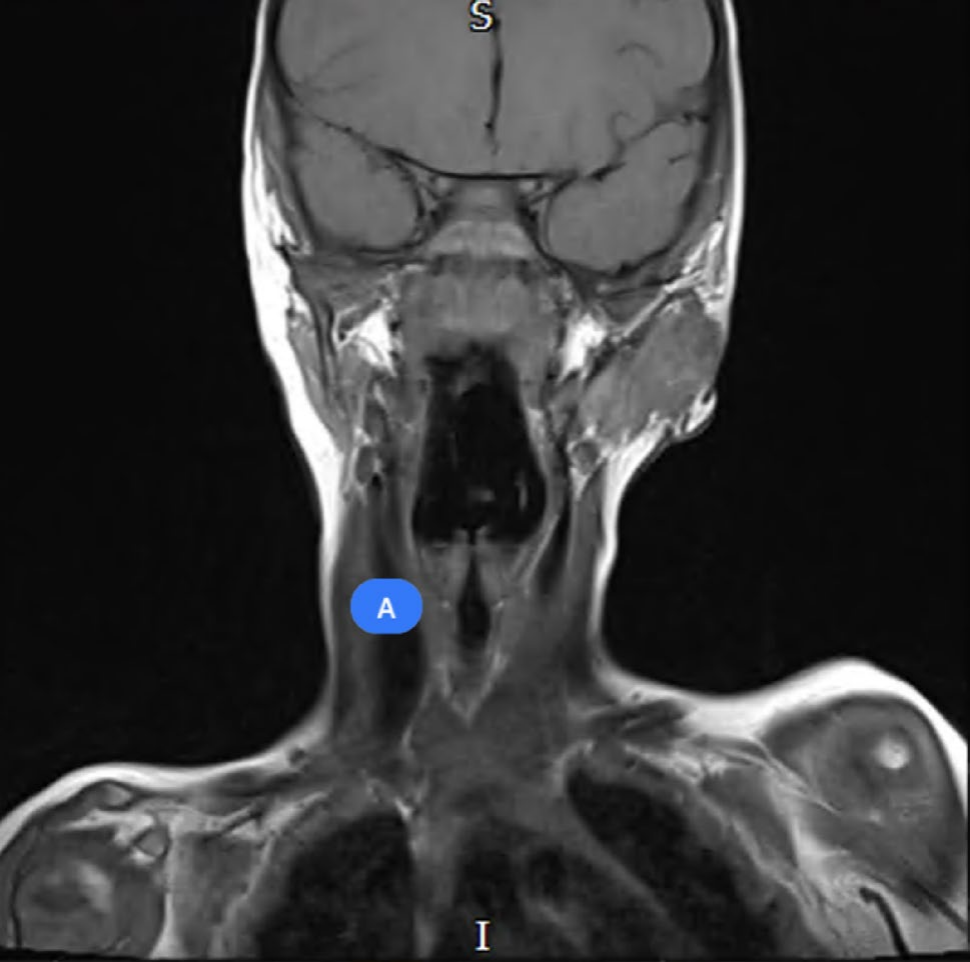
Pre-operative MRI imaging of left first branchial arch cleft cyst

**Fig. 16:**
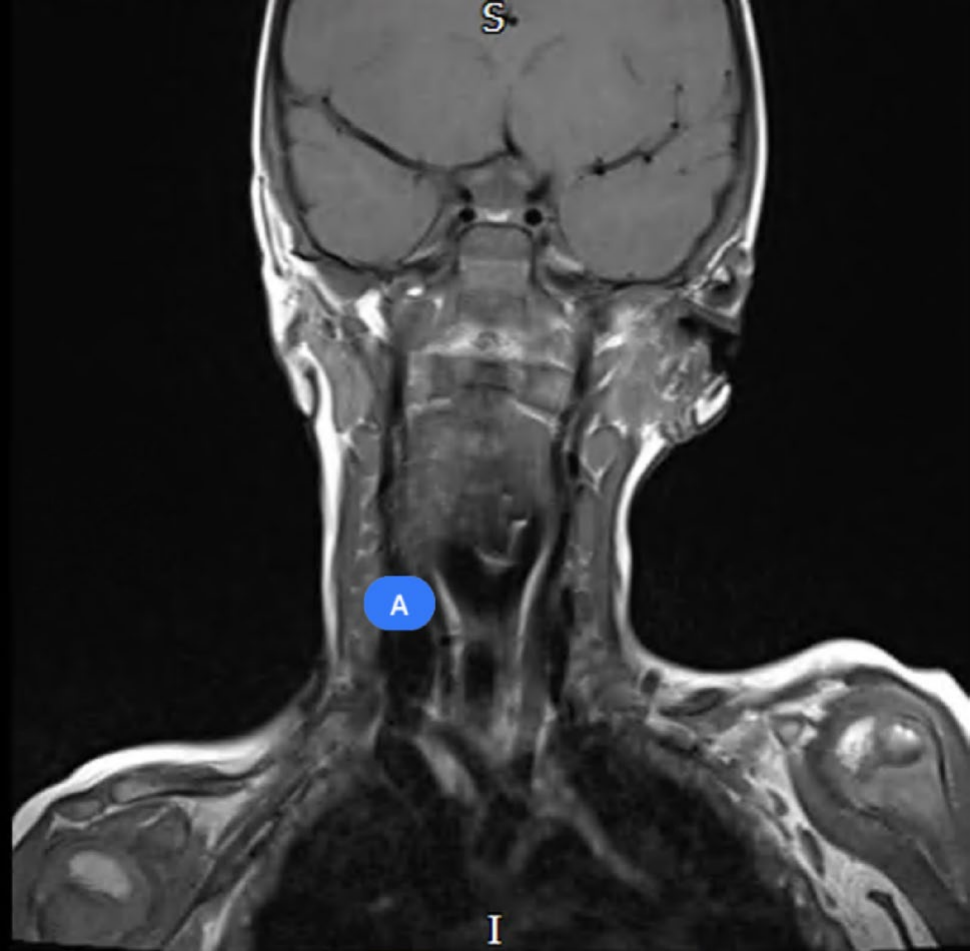
Pre-operative MRI imaging of left first branchial arch cleft cyst

Follow-up durations varied substantially, with short intervals of four weeks common, while one patient was followed for twenty-two months. The most common timing of the first follow up appointment was within 4–6 weeks of the operation date with the histopathology results. Most common prescription on the first clinic appointment was a healing oil/ointment to promote optimal skin closure. One outlier case had a six-year interval between clinic presentation and surgical intervention, highlighting heterogeneity in care pathways. Looking into this case further, the delay was mainly attributed to COVID-19 related reasons as well as reluctance of the family to proceed with an operation.

**Table 1:** Results of retrospective study

Category	Findings
Patients	11 over 10 years
Age at presentation	Mean: 5.55 years; Median: 4 years; Range: 2–13 years
Clinical presentation	Persistent sinus: 10/11 (91%) • With purulent discharge: 9/11 (82%) • Swelling only: 1/11 (9%)
Laterality	Left-sided: 8/11 (73%) Right-sided: 3/11 (27%)
Imaging used	MRI: 8/11 (73%) USS: 2/11 (18%) Repeat MRI: 1 patient
Intraoperative findings	Tract to external auditory canal: 9/11 (82%) Tract from level I: 1/11 (9%) Tract from level III: 1/11 (9%)
Postoperative outcomes	No complications: 5/11 (45%) Wound infection: 3/11 (27%) Marginal mandibular weakness: 2/11 (18%, full recovery) Tragal numbness: 1/11 (9%)
Recurrence	1 patient (revision excision at tertiary centre, resolved)
Follow-up	Range: 4 weeks – 22 months Common: 4–6 weeks with histopathology review Longest delay to surgery: 6 years (COVID/family reluctance) Most common treatment: healing oil/ointment

**Table 2:** Results of retrospective study

Patient	Age (yrs)	Presentation	Laterality	Imaging	Fistula/Tract Course	Post-op Complications	Facial Nerve Weakness	Recurrence	Follow-up
1	2	Purulent sinus	Left	MRI	Level II → ear canal	None	None	None	4 weeks
2	3	Purulent sinus	Left	MRI	Level II → ear canal	Wound infection	None	None	4 weeks
3	4	Purulent sinus	Right	MRI	Level II → ear canal	None	None	None	6 months
4	5	Purulent sinus	Left	MRI	Level I	None	None	None	3 months
5	5	Purulent sinus	Left	MRI	Level II → ear canal	Wound infection	Transient	Yes	22 months
6	6	Purulent sinus	Left	Ultrasound	Level II → ear canal	None	None	None	4 weeks
7	7	Purulent sinus	Right	MRI	Level III	Marginal mandibular weakness (resolved)	Yes – resolved	None	4 weeks
8	8	Swelling	Left	MRI	Level II → ear canal	None	None	None	6 months
9	9	Purulent sinus	Left	MRI	Level II → ear canal	Wound infection	None	None	2 months
10	12	Purulent sinus	Right	Ultrasound	Level II → ear canal	Marginal mandibular weakness (resolved)	Lip asymmetry on crying – resolved	None	4 weeks
11	13	Purulent sinus	Left	MRI	Level II → ear canal	Tragal numbness	None	None	4 weeks

## Discussion

Branchial anomalies represent a rare but important group of congenital head and neck conditions in the paediatric population. Their spectrum of presentation is broad, ranging from recurrent sinus discharge to isolated swelling, and this variability often leads to diagnostic uncertainty and delayed intervention. The rarity of these anomalies is compounded by their intimate anatomical relationship to the parotid gland and facial nerve, which adds further complexity to management [3, 4]. Existing literature highlights wide variation in reported outcomes, with surgical morbidity and recurrence rates largely influenced by the extent of the tract and the precision of preoperative imaging [5]. In recent years, MRI has emerged as the preferred modality, offering superior delineation of tract anatomy compared to ultrasound, which was more commonly used in earlier cohorts [1, 2]. Within this context, the present series adds to the evidence base by characterising the clinical course, imaging approaches, operative findings, and postoperative outcomes observed across a decade of experience.

Accurate diagnosis is complicated by the overlap of clinical features with other congenital and acquired pathologies, such as pre-auricular sinuses, epidermoid inclusion cysts, or suppurative infections in the parotid region. Delayed or incorrect diagnosis can lead to recurrent infection, abscess formation, and scarring, all of which may complicate definitive surgical management. Furthermore, operative excision of these lesions carries inherent risks, particularly with regard to potential injury to the facial nerve [6].

Over the last several decades, classification systems have been proposed to describe first branchial cleft anomalies. Work by Work and Arnot distinguished type I lesions, which represent ectodermal duplications of the external auditory canal, from type II lesions, which are thought to include both ectodermal and mesodermal elements and extend towards the angle of the mandible or submandibular region. This classification has been revised and enriched since its publication [7, 8]. In clinical practice, however, lesions often show variable courses, and the precise preoperative classification is less important than careful delineation of their anatomical relation to critical structures.

Although isolated reports and small case series exist in the literature, comprehensive paediatric data remain sparse. Most published work has emphasised the rarity of these lesions, the diversity of their clinical presentation, and the operative challenges they pose. Rates of facial nerve injury, recurrence, and postoperative wound infection vary considerably between series, reflecting both small sample sizes and differences in diagnostic and surgical approaches.

This case series highlights the presentation and outcomes of first branchial cleft anomalies in a UK paediatric tertiary centre. Although limited in size, it offers insight into clinical presentation, diagnostic practices, operative findings, and surgical complications over a decade. Our findings both reinforce established knowledge and raise important questions for future research.

### Clinical presentation and diagnostic challenges

The overwhelming majority of our patients presented with a persistent discharging sinus, most often purulent, which is consistent with previous descriptions of first branchial cleft anomalies. In the literature, presentation is often delayed because lesions may mimic recurrent abscesses or infected pre-auricular sinuses [9, 10]. Early misdiagnosis can result in repeated incision and drainage procedures or prolonged courses of antibiotics, which may complicate subsequent definitive surgery through scarring and distortion of tissue planes [11].

**Fig. 17:**
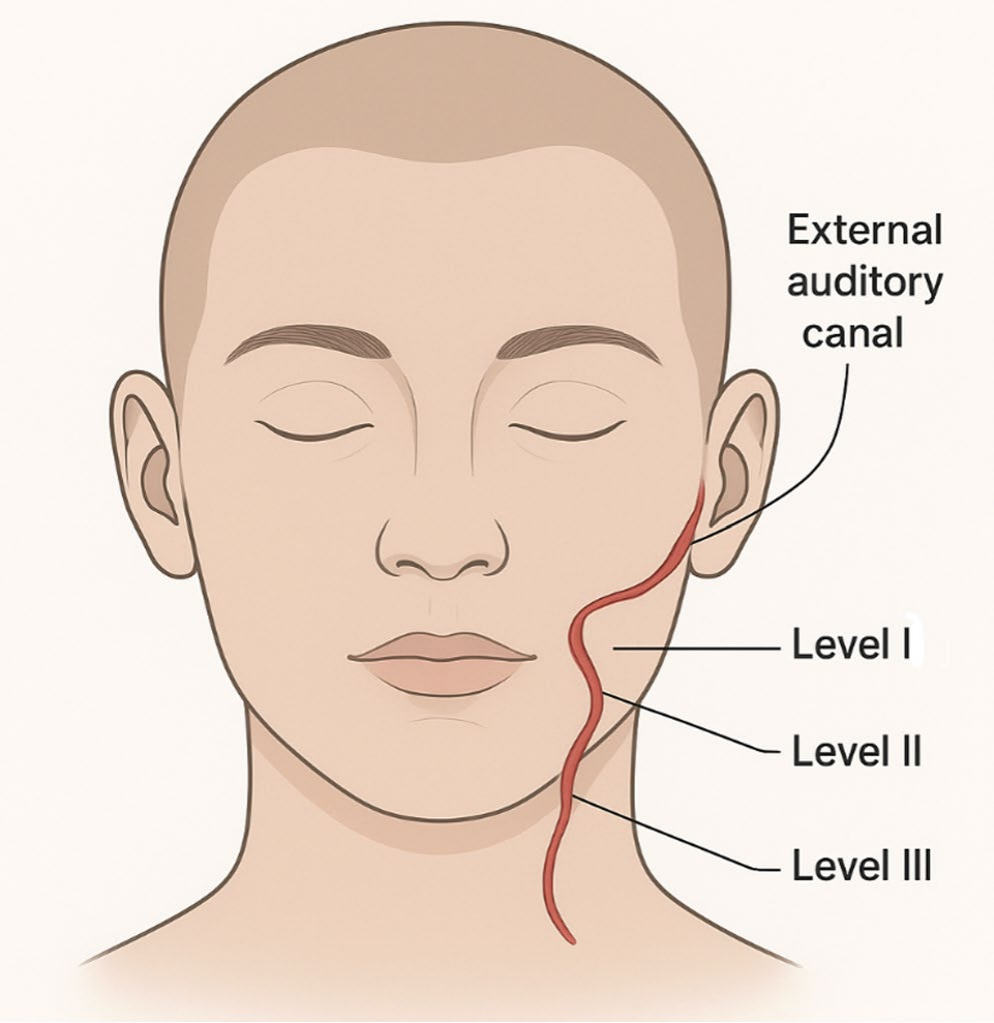
Courses of first branchial cleft fistulas in our study

Interestingly, eight of our eleven cases were left-sided. While there is no established embryological explanation for lateral predominance, several other small series have also reported more left-sided lesions, whereas larger reviews often show an equal distribution. It remains uncertain whether this represents biological variation, reporting bias, or chance given the small numbers in most studies. The different courses of the first branchial cleft fistulas are demonstrated in Fig. 17.

One of our cases presented only as swelling without discharge, illustrating the variable clinical spectrum. Pure cystic forms may remain asymptomatic until secondarily infected. A high index of suspicion is required in such cases, particularly when the lesion is located near the external auditory canal, the parotid region, or the angle of the mandible. It is challenging to differentiate these lesions from a persistent lymph node, a pre-auricular cyst or an epidermoid cyst and if treated inappropriately, they might recur due to incomplete excision of the embryological remnant [12]. It is therefore suggested to have a high level of suspicion and consider imaging for cases that do not appear simple, to avoid incomplete excision and recurrence.

### Imaging and preoperative planning

MRI was the most frequently used imaging modality, consistent with its superiority in demonstrating soft tissue anatomy and defining tract course relative to the parotid gland and facial nerve. In the paediatric population, MRI avoids ionising radiation but may require sedation or even general anesthetic in younger children. The ability of MRI to reveal extension into the external auditory canal and to define whether the tract passes superficial or deep to the facial nerve is invaluable for surgical planning [13]. Occasionally, even the MRI can be difficult to interpret, so intraoperative facial nerve monitoring is commonly used to ensure the facial nerve remains intact. In addition, one should be alert while dissecting due to toe complexity of the embryological tissues in order to follow adequately the possible tract to ensure complete excision regardless the MRI findings. An intraoperative use of contrast/dye is also suggested in some cases to follow the tract adequately [14, 15].The reliance on MRI in our centre is consistent with international trends, although some centres continue to use CT, particularly in adults.

Ultrasound was used in two cases, possibly reflecting earlier diagnostic workup or limitations of MRI availability. While ultrasound can identify cystic lesions, its capacity to demonstrate deep tract anatomy is limited. In our experience, MRI provided critical operative guidance in the majority of cases. The absence of CT imaging in our cohort reflects current practice in children, where radiation avoidance is a priority.

In summary, preoperative imaging is particularly important in first branchial cleft anomalies compared to second cleft anomalies because of the intimate relationship with the facial nerve. Reports in the literature emphasise that inadequate delineation of the tract increases the risk of nerve injury and recurrence. Our findings support the view that MRI offers the best balance of diagnostic clarity and safety in children.

### Operative findings and surgical considerations

Nine of eleven tracts coursed from level II of the neck to the external auditory canal. This pattern corresponds most closely with type II lesions in the Work classification, which involve both ectodermal and mesodermal elements and often extend to the cartilaginous external auditory canal. The predominance of type II anomalies in published series is well described, whereas pure type I lesions are much less common.

The frequent involvement of the parotid region underscores the risk to the facial nerve. The marginal mandibular branch is particularly vulnerable during surgical dissection at the angle of the mandible [16]. In our series, two patients developed postoperative marginal mandibular weakness, but both deficits were transient and resolved completely. This aligns with published recurrence of transient weakness rates ranging from 7 to 20%, and persistent weakness in 1 to 5% of cases [4, 17]. Several studies provide useful benchmarks for our findings. Olsen et al. reported on 40 cases, noting facial nerve palsy in 5%, recurrence in 12%, and infection in 15% [18]. Triglia et al. described 39 paediatric cases with a recurrence rate of 10% [19]. Compared with these series, our cohort showed similar transient facial nerve weakness rates. The absence of persistent deficits in our cohort is encouraging but must be interpreted with caution given small numbers.

Facial nerve monitoring is recommended by many authors during surgery for first branchial cleft anomalies. Its use was also consistently documented in our dataset. Incorporating routine intraoperative monitoring, particularly in revision or complex cases, may help mitigate risk and should be considered standard practice.

### Postoperative complications and recurrence

Wound infection occurred in three patients (27%). This is somewhat higher than reported in many series, where rates range from 10 to 20%. The high prevalence of chronically infected sinuses at presentation may have predisposed to infection. Optimising infection control preoperatively, including delaying surgery until active infection resolves, may reduce postoperative morbidity. In addition, antibiotic use intra-operatively without any specific concern is not supported in the literature and should be avoided not to result in antibiotic resistance [20, 21].

Recurrence was explicitly documented in one patient. Published recurrence rates vary widely, from less than 5% to over 20%, often reflecting incomplete excision or distortion of anatomy by previous infection or intervention. El Omri et al. present a case series with a thorough literature review identifying a recurrence rate of up to 20% if the operation is completed during an acytely infected phase. Percentages drop to 5% if the operation is performed on a cold not infected setting [22]. Revision surgery is more complicated and encounters more risks regarding facial nerve injury due to dissection into scarred tissues. According to the literature, it occasionally involves tracking down the fistula remnants towards the thyroid gland and in rare cases proceed with a hemithyroidectomy or explore the submandibular gland due to high proximity of the lesions [23]. Our results highlight the importance of systematic and standardised follow-up in order to capture accurate recurrence rates.

### Limitations of the study

The principal limitation is the small sample size, which precludes statistical inference and limits generalisability. Retrospective design and reliance on free-text documentation could result to mis-interpreted data. Follow-up duration was short in many patients, which might underestimates recurrence rates. Finally, as a single-centre study, findings may not reflect broader practice patterns due to single surgeon bias or single ENT team policies and guidelines.

### Implications for clinical practice

Despite these limitations, our study has important implications. First, clinicians should maintain suspicion for first branchial cleft anomalies in children presenting with recurrent discharging sinuses near the angle of the mandible or parotid region. Second, MRI should be considered the imaging modality of choice for preoperative planning. Third, counselling of families should include discussion of wound infection risk and the small but real possibility of facial nerve weakness, even if transient. Finally, systematic follow-up with documentation of recurrence and nerve function is essential for ongoing audit and quality improvement.

### Future research directions

Future work should focus on collaborative multi-centre registries to accumulate larger datasets. Prospective design with standardised data collection—including preoperative imaging, operative technique, intraoperative nerve monitoring, facial nerve assessment, and follow-up—would allow more robust analysis of outcomes. Given the rarity of first branchial arch cleft cysts and fistulas, it is useful to create a multi-centre database to accumulate reliable data to further characterise this pathology. This will enable to treat the condition more appropriately, will lead to more accurate diagnosis and less complications or recurrence rates. Further study of embryological variants and potential genetic associations may also shed light on pathogenesis. Finally, economic evaluation of imaging strategies and operative approaches could guide best use of resources in paediatric otolaryngology services.

## Conclusion

This ten-year, single-centre case series offers a window into the diverse ways first branchial cleft anomalies present and are managed in children. Although uncommon, these lesions often make themselves known through a persistently discharging sinus at the angle of the mandible, with a striking tendency to occur on the left side. MRI emerged as the investigation of choice, not only confirming the diagnosis but also guiding safe surgical planning by clarifying the course of the tract in relation to the parotid gland and facial nerve.

Surgery provided definitive treatment in the majority of cases, though not without risk. Wound infection was the most frequent complication, and a minority of children developed temporary marginal mandibular weakness, but reassuringly, no child sustained permanent facial nerve dysfunction. A single recurrence was observed and was revised successfully in another tertiary paediatric ENT centre.

These observations emphasise several important messages for clinical practice. Careful preoperative imaging, meticulous surgical technique, and structured postoperative surveillance are all crucial to minimise complications and reduce the risk of recurrence. Families should be counselled appropriately about potential short-term complications while reassured regarding the very low likelihood of lasting nerve injury.

In summary, this series contributes to the wider body of literature by reinforcing consistent patterns of presentation and management. It also points to ongoing gaps in knowledge—particularly regarding recurrence rates, the potential value of intraoperative nerve monitoring, and the long-term course of these children. Addressing these questions will require prospective, multi-centre collaboration to generate the kind of robust data that can guide evidence-based care for this challenging but treatable anomaly.

## Data Availability

No datasets were generated or analysed during the current study.
